# Dolly in the Hospital

**Published:** 1886-11-13

**Authors:** 


### DOLLY IN THE HOSPITAL.

**Children and Toys.** How cheerless would this world be without children, and how cheerless would be the children without their toys! Before the bitter realities of life thrust themselves upon us, the natural impulse of the mind is to seek pleasure in that which surrounds it, and this impulse finds expression in play. "All work and no play makes Jack a dull boy;" but it does a great deal more, for it dwarfs and stunts the natural joyousness of the child and prematurely ages it. Let us look at the poor little waif who is forced to work or to beg while it is yet a mere babe, and we shall soon see upon its face something of the effects which the denial of play produces. Play has also its curative qualities. The pleasures which it excites divert the mind and dissipate its moody burden. The little sick child in the hospital (unless its illness demands absolute quietude) requires the diversion of amusement as much as the little one who is well; and in many cases, the doctors will tell us, a toy will actually facilitate recovery, for it will make the child forget its illness in its play.

**The Santa Claus Society.** Some kind ladies organised, about a year or so ago, an association, which they called the Santa Claus Society, the main object of which is to provide children in hospitals with dolls and other toys at Christmas-time. And who was Santa Claus? some of my younger readers may be asking. Santa Claus, or Klaus, is merely the Dutch name of good St. Nicholas, the patron saint of boys. In Flanders, in Holland, and in several parts of Germany, the little children, just before Christmas, will, to this day, put out their shoes or stockings before retiring to rest, in the belief that Santa Claus will surreptitiously place therein a toy, as a reward for their good conduct. The mystery which surrounds the advent of a present is sure to enhance the pleasure it affords. When the little child sees, on awakening in the morning, a dainty little parcel or a pretty box on its coverlet, there are sure to be ample indications of delighted surprise. With the memory of fairy stories still fresh within her mind, the little girl will hardly be inclined to doubt your word when you suggest that dolly must have come down the chimney; for though there is no soot upon her pretty frock, the guardian elves, she thinks, may easily have protected dolly in her adventurous journey to her mistress.

**An Opportunity for Christmas.** The distribution of the dolls and toys by the ladies of the Santa Claus Society last Christmas, among the children who then chanced to be within the walls of some six or seven of the poorer London hospitals, caused intense delight, and, if all I hear is correct, there are similar good things in store for the coming Yuletide. The delight with which they unpacked their parcels, could the givers have been present, would have been ample repayment for their pains. The dolls, which numbered over a hundred, varied much in kind—mamma dolls and baby dolls, boy dolls and girl dolls, crying dolls and talking dolls, black dolls and a lotus-eyed beauty from Japan, all found their enamoured admirers. The great charm to the girls was of course that they could undress them; and many of the donors had been so thoughtful as to provide little dolly with a nightdress, so that she might suitably take her rest with her youthful mistress. The little children at the East London Hospital at Shadwell were overjoyed at their beautiful presents, for toys are few and far between amongst the children of the poor. One little girl trotted down her ward with her new baby doll to a chair, which she dragged with much difficulty towards the fire. When she had arranged matters conformably to her own little mind, she clambered up, and, after informing her "dear dolly" that she was going to undress her "in the warm," proceeded to suit the action to the word, and that, too, with true motherly tenderness.

**Sceptical Boys!** Most of the little ones seemed to have full faith in the reality and beneficence of good Santa Claus, except some of the boys, who evinced a certain scepticism on the subject. One little fellow simply refused to believe that the children's patron saint had power to descend the chimney; and to enforce his contention, he pointed out that his toys were "quite clean and had no soot on them"; and another astute youngster said he had looked at the floor of the ward

"quite early" on Christmas morn, and had seen no black footmark on it!

The Modern Santa Claus.

The modern Santa Claus is undoubtedly Mr. Henry Labouchere, M.P., the proprietor and editor of *Truth*. To his kindly thought and action is no doubt due the formation of the Santa Claus Society. For several years he has organised at Christmas a "*Truth* Toy Fund" for the benefit of all the children to be found in the hospitals and workhouses of London. Last year, through Mr. Labouchere's agency, over 13,000 children had each a toy given them at Christmas, and one hundred and fifteen large toys were also presented for general use in the various institutions. This Christmas, no doubt, many more children will be found awaiting with pleasurable expectation the visit of their good friend Mr. Labouchere, although he is only known to them as Santa Claus. All who have children should send something to the "*Truth* Toy Fund," Truth Buildings, Queen Anne's Mansions, S.W., for the poor children in the various London hospitals, workhouses, workhouse infirmaries, and workhouse schools.

